# Survival and prognostic factors analysis of carbon ion radiotherapy for limited-stage small cell lung cancer

**DOI:** 10.3389/fonc.2026.1898779

**Published:** 2026-10-01

**Authors:** Xuexue Liang, Qinli Gong, Yihe Zhang, Ling Tian, Xiaoli Zhao, Bole Wang, Zhenglin Li, Pengqing Li, Yancheng Ye, Yanshan Zhang

**Affiliations:** 1 School of Public Health, Gansu University of Chinese Medicine, Lanzhou, Gansu, China; 2 Department I, Radiation Therapy Center, Gansu Wuwei Tumour Hospital, Wuwei, Gansu, China; 3 Follow-up Office, Gansu Wuwei Tumour Hospital, Wuwei, Gansu, China

**Keywords:** carbon ion radiotherapy, limited-stage small cell lung cancer, prognostic factors, safety, survival analysis

## Abstract

**Objective:**

To investigate the clinical efficacy, safety and prognostic influencing factors of carbon ion radiotherapy (CIRT) in patients with limited-stage small-cell lung cancer (LS-SCLC).

**Methods:**

The clinical data of 39 patients with LS-SCLC who received CIRT from June 2020 to August 2026 were retrospectively analyzed. Survival outcomes and adverse reactions were evaluated, and Cox regression was used to analyze prognostic factors.

**Results:**

All patients completed the treatment. The 3-month objective response rate (ORR) was 64.10%. The 1- and 2-year local control rates were both 91.2%. The 1- and 2-year overall survival (OS) rates were 91.65% and 62.45%, with a median OS of 44 months. The 1- and 2-year progression-free survival (PFS) rates were 72.00% and 47.77%, with a median PFS of 24 months. Serum levels of NSE, CYFRA21-1, SCC and CEA decreased significantly after treatment. Acute treatment-related adverse reactions were mainly grade I-II and could be relieved after symptomatic treatment. Stratified Cox regression indicated that KPS score was an independent protective factor for OS. Univariate analysis suggested that the magnitude of post-treatment reduction in SCC and CYFRA21-1 was potentially associated with survival outcomes.

**Conclusion:**

CIRT achieves favorable local tumor control with low acute radiotherapy-related toxicity, and can serve as an alternative therapeutic option for LS-SCLC. This is a single-center, small-sample retrospective study, and the conclusions regarding efficacy and prognosis need to be further verified in larger-sample cohorts.

## 1 Introduction

Small-cell lung cancer (SCLC) is a highly aggressive and rapidly progressive neuroendocrine tumor, accounting for approximately 15%-20% of all lung cancers (1, 2). Limited-stage SCLC (LS-SCLC) is defined as tumor confined to one-sided thoracic cavity, mediastinum and supraclavicular lymph nodes without distant metastasis. Its standard treatment consists of concurrent chemoradiotherapy plus consolidation therapy (3). Although LS-SCLC is highly sensitive to initial chemoradiotherapy, conventional regimens such as etoposide-platinum-based chemotherapy combined with thoracic radiotherapy can achieve prominent short-term therapeutic effects. Nevertheless, the risk of disease recurrence and metastasis remains high (4), with a 5-year overall survival rate of merely 20%-30% (5), resulting in unsatisfactory long-term prognosis for patients.

Conventional photon radiotherapy serves as a core therapeutic modality for LS-SCLC, yet it has limitations in physical dose distribution. When escalating the dose to the tumor target volume, it tends to injure normal organs including the lung, esophagus and heart. This leads to a relatively high incidence of adverse events such as radiation pneumonitis and radiation esophagitis, which impair patients’ treatment tolerance and quality of life (6). As a heavy-ion beam, carbon ion possesses unique physical and biological advantages. It exhibits the Bragg-peak dose-distribution property, which enables high-dose deposition within the tumor target and markedly spares surrounding normal tissues (7). Meanwhile, it has a high relative biological effectiveness (RBE), and can exert potent cytotoxic effects even on photon-resistant tumor cells (8). Given these merits, carbon-ion radiotherapy has shown great promise for multiple solid tumors. Nevertheless, more clinical evidence is still required to support its application in LS-SCLC. Therefore, this study retrospectively analyzed clinical data of LS-SCLC patients treated with carbon-ion radiotherapy, aiming to provide evidence for its clinical application.

## 2 Materials and methods

### 2.1 Study subjects

Thirty-nine patients with small-cell lung cancer who received carbon-ion radiotherapy at Gansu Wuwei Cancer Hospital from June 8, 2020 to August 13, 2026 were enrolled.

#### 2.1.1 Inclusion criteria

1. Histologically or cytologically confirmed diagnosis of small-cell lung cancer;
2. Age ≥18 years old with a KPS score ≥70;
3. Meeting the tolerance criteria for radiotherapy, detailed requirements are as follows:
  - Bone marrow function: peripheral white blood cell count ≥3.0×10^9^/L, platelet count ≥100×10^9^/L;
  - Hepatic and renal function: alanine aminotransferase, aspartate aminotransferase, serum creatinine and urea nitrogen ≤2.5 × upper limit of normal (ULN);
  - Cardiopulmonary function: no severe pulmonary ventilation dysfunction or other important organ dysfunction that would preclude radiotherapy;
4. Limited-stage lesions without extensive distant metastasis and without massive malignant pleural/pericardial effusion;
5. No prior history of thoracic radiotherapy and no history of other malignant tumors.

#### 2.1.2 Exclusion criteria

① Presence of extensive distant metastasis;

② Presence of active infection, severe bleeding tendency or other contraindications to radiotherapy;

③ Patients with incomplete follow-up data or unwillingness to cooperate with follow-up.

This study was approved by the Medical Ethics Committee of our hospital (No. 2026-LLSC-69). All patients were informed and provided written informed consent.

### 2.2 Treatment methods

#### 2.2.1 Positioning and target delineation

All enrolled patients were histopathologically confirmed to have small-cell lung cancer. Contrast-enhanced chest computed tomography (CT), positron emission tomography-computed tomography (PET-CT), and contrast-enhanced brain magnetic resonance imaging (MRI) were performed before treatment. The diagnosis of limited-stage small-cell lung cancer (LS-SCLC) was established based on the above imaging findings. Laboratory examinations included blood routine, hepatic and renal function, electrolytes, and coagulation function. Serum tumor markers including NSE, CYFRA21-1, CEA, SCC and ProGRP were officially available in our hospital since July 2024; therefore, complete panel of these markers could only be obtained for patients enrolled after July 2024. The Karnofsky performance-status (KPS) score was recorded. Pulmonary function test and electrocardiogram were also completed to evaluate patients’ tolerance to concurrent chemoradiotherapy.

Patients were placed in the supine or prone position according to tumor location, beam entry direction and respiratory-motion characteristics. The patients placed their hands above the head in a crossed manner with the left hand on top, and a pillow was placed under the occiput. A large vacuum cushion combined with thermoplastic mask was used for dual-modality immobilization. Simulation CT scans were performed on a Siemens SOMATOM-Definition AS scanner with a slice thickness of 3 mm, including non-contrast, contrast-enhanced sequences and 4D-CT respiratory-gated scans. Acquired CT images were imported into the rtstation system for target contour delineation (9). Contours were exported in DICOM-RT format and subsequently imported into the ciPlan 1.0 treatment-planning system for further plan design.

The gross tumor volume (GTV) refers to radiologically-visible primary tumor and involved lymph nodes; the clinical tumor volume (CTV) includes the GTV plus lymph-drainage regions (ipsilateral hilum, subcarinal and mediastinum). The internal target volume (ITV) is defined as CTV plus the range of 4D-CT maximum-intensity projection (MIP) (10). A simultaneous integrated boost (SIB) dual-target plan was adopted to deliver irradiation to two targets within each fraction: planning-target volume PTV1: ITV expanded by 0.5 cm; PTV2: GTV plus MIP-derived range expanded by 0.5 cm. Prescription doses: PTV1: 48 Gy (RBE)/12fx, 4 Gy (RBE)/fx; PTV2: 24 Gy (RBE)/12fx, 2 Gy (RBE)/fx. Prophylactically-irradiated lymph-drainage regions received PTV1 only, with a cumulative dose of 48 Gy (RBE)/12 fx, 5 fractions per week. The gross tumor volume (including primary tumor and metastatic lymph nodes) received superimposed irradiation from both PTV1 and PTV2 to a cumulative dose of 72 Gy (RBE)/12fx. The planning constraint required that 90 % of the PTV volume receive ≥95 % of the prescription dose. Organs-at-risk (OARs) including lungs, esophagus, spinal cord and heart were contoured simultaneously (11). Normal-tissue dose limits referred to the Chinese Ion Therapy Guidelines (2020 edition) formulated by the Chinese Society for Radiation Oncology, Chinese Medical Doctor Association (12): spinal cord Dmax<45 Gy (RBE), heart Dmax<72 Gy (RBE), lung Dmean<14 Gy (RBE), unilateral-lung V20Gy (RBE)<20 %, V5Gy (RBE)<40 %, esophagus Dmax<60 Gy (RBE). In clinical practice, safety constraints for organs-at-risk took priority, and the actual irradiation dose could be individually reduced according to tumor anatomical relationship, patient tolerance and tumor shrinkage during treatment.

#### 2.2.2 Treatment-planning design

The domestically-produced carbon-ion treatment-planning system (ciPlan 1.0) was adopted. The relative biological effectiveness (RBE) was calculated using the mixed-beam LQ model (MBM LQ). LQ-model parameters were obtained based on HSG-cell-survival experiments, and the RBE depth-distribution curve at the 10 % cell-survival level was calculated for range-modulator design and radiotherapy-plan optimization. To achieve uniform distribution of biologically-effective dose within the planning target volume, the planning system adopted an iterative optimization algorithm to solve the irradiation-dose weight for each carbon-ion Bragg peak, and the physical-dose distribution was output according to the weight of each Bragg peak. Consistent with the Japanese MKM model, the RBE model of Wuwei ciPlan follows the formulas: Clinical dose = biologically-effective dose × 1.43; biologically-effective dose = physical absorbed dose × RBE. Under the same clinical-dose condition, the physical-absorbed-dose difference between the MKM model and ciPlan is small (12, 13). All treatment plans were transmitted and executed via the ciTreat V2.0 system. The carbon-ion beam energy ranged from 120 to 400 MeV/u, and either passive-scanning or spot-scanning technique was applied.

The comprehensive treatment regimen included concurrent and sequential platinum-etoposide-based chemotherapy: etoposide 100 mg/m^2^ on days 1-3 plus cisplatin 75 mg/m^2^ on day 1, with a 21-day cycle for a total of 4 cycles. For patients with cisplatin intolerance or renal dysfunction, cisplatin was replaced by carboplatin (AUC 5-6 on day 1) upon clinical physician’s evaluation. Concurrent chemotherapy was initiated in the same week when carbon-ion radiotherapy started; 1-2 cycles of concurrent chemotherapy were delivered during the radiotherapy course, and the remaining cycles were supplemented after the completion of radiotherapy. The remaining patients received induction plus consolidation sequential chemotherapy. Prophylactic cranial irradiation (PCI) was administered to patients who achieved complete or partial response after radiotherapy, without intracranial metastasis and with tolerable physical status, at a prescription dose of 25 Gy (RBE)/10 fractions.

### 2.3 Observation indicators

#### 2.3.1 Efficacy evaluation

Short-term therapeutic efficacy was evaluated by contrast-enhanced chest CT at 3 months after the completion of treatment. According to the Response Evaluation Criteria in Solid Tumors (RECIST version 1.1), responses were classified as complete response (CR), partial response (PR), stable disease (SD), and progressive disease (PD). The objective response rate (ORR) = (number of CR + PR cases)/total number of cases × 100%. Local control failure was defined as the progression of primary tumor and involved lymph nodes within the irradiation field; distant metastasis outside the target volume was not counted as local failure. The local control rate (LC) was calculated from the end of carbon-ion radiotherapy using the Kaplan-Meier method. Patients without local-control-failure events were censored at the time of the last valid follow-up.

#### 2.3.2 Survival indicators

Follow-up was performed via outpatient re-examination and telephone interview. The starting point of follow-up was the initiation date of treatment, and the endpoints were disease progression or death. Progression-free survival (PFS) was defined as the time from treatment initiation to the first occurrence of disease progression or death from any cause. Overall survival (OS) was defined as the time from treatment initiation to death from any cause or the last follow-up. Patients lost to follow-up were censored at the date of last contact.

#### 2.3.3 Safety evaluation

Adverse events were recorded during treatment and within 3 months after treatment according to the Common Terminology Criteria for Adverse Events (CTCAE, version 5.0), including radiation-induced esophagitis, radiation-induced pneumonitis, nausea and vomiting, hematological toxicity and others. The severity was graded from grade I to IV.

#### 2.3.4 Statistical methods

Data analyses were performed using SPSS 26.0, GraphPad Prism 10 and R 4.6.1 software. Count data were presented as number of cases (percentage). The Kaplan-Meier method was used to plot survival curves, and the Log-rank test was applied for between-group comparison. The Cox proportional-hazards regression model was adopted to analyze independent prognostic factors. A P-value < 0.05 was considered statistically significant.

## 3 Results

### 3.1 Baseline characteristics of patients

A total of 39 patients with limited-stage small-cell lung cancer were enrolled in this study, including 31 males (79.5 %) and 8 females (20.5 %). The median age was 61 years (range, 25-82 years). There were 20 patients (51.3 %) with a KPS score of 90, 18 patients (46.2 %) with 80, and 1 patient (2.5 %) with 70; 21 patients (53.8 %) had a smoking history. T4 was the predominant T-stage (16 cases, 41.0 %). N2 (15 cases, 38.5 %) and N3 (17 cases, 43.5 %) accounted for the majority of N-stages. Stage IIIB was the most frequent clinical stage (17 cases, 43.5 %). Twenty-five patients (65.8 %) received concurrent chemoradiotherapy, 13 patients (34.2 %) received sequential chemotherapy, and 1 patient declined chemotherapy. Thirty-one patients (81.6 %) completed 4-6 cycles of chemotherapy, and 23 patients (59.0 %) received immunotherapy. The cohort was predominantly elderly, male and locally-advanced, which was consistent with the clinical epidemiological characteristics of limited-stage small-cell lung cancer (**Table 1**).

**Table 1:** Baseline characteristics of patients.

Characteristic	N (%)	Characteristic	N (%)
Gender		Smoking	
Male	31 (79.5)	Yes	21 (53.8)
Female	8 (20.5)	No	18 (46.2)
Age		T-stage	
Mean (standard deviation)	59.51 ± 12.39	T1	6 (15.4)
KPS		T2	7 (17.9)
70	1 (2.5)	T3	10 (25.7)
80	18 (46.2)	T4	16 (41.0%)
90	20 (51.3)	Clinical stage	
N-stage		I B	1 (2.6)
N0	4 (10.3)	II B	4 (10.3)
N1	3 (7.7)	III A	6 (15.4)
N2	15 (38.5)	III B	17 (43.5)
N3	17 (43.5)	III C	11 (28.2)
Chemoradiotherapy sequence		Immunotherapy	
Sequential	13 (34.2)	No	16 (41.0)
Concurrent	25 (65.8)	Yes	23 (59.0)

One patient declined chemotherapy.

### 3.2 Clinical efficacy

All the 39 patients completed carbon-ion radiotherapy. The 3-month objective response rate (ORR) was 64.10% (25/39), including 7 cases (17.9%) of complete response (CR), 18 cases (46.2%) of partial response (PR), and 14 cases (35.9%) of stable disease (SD). The 1-year ORR was 47.37% (9/19). At the last follow-up, 6 patients developed disease progression and 13 patients died. The 1- and 2-year progression-free survival (PFS) rates were 72.00% and 47.77%, respectively, with a median PFS of 24 months. The 1- and 2-year overall survival (OS) rates were 91.65% and 62.45%, respectively, with a median OS of 44 months. Both the 1- and 2-year local control (LC) rates were 91.2% (**Figure 1**).

**Figure 1:**
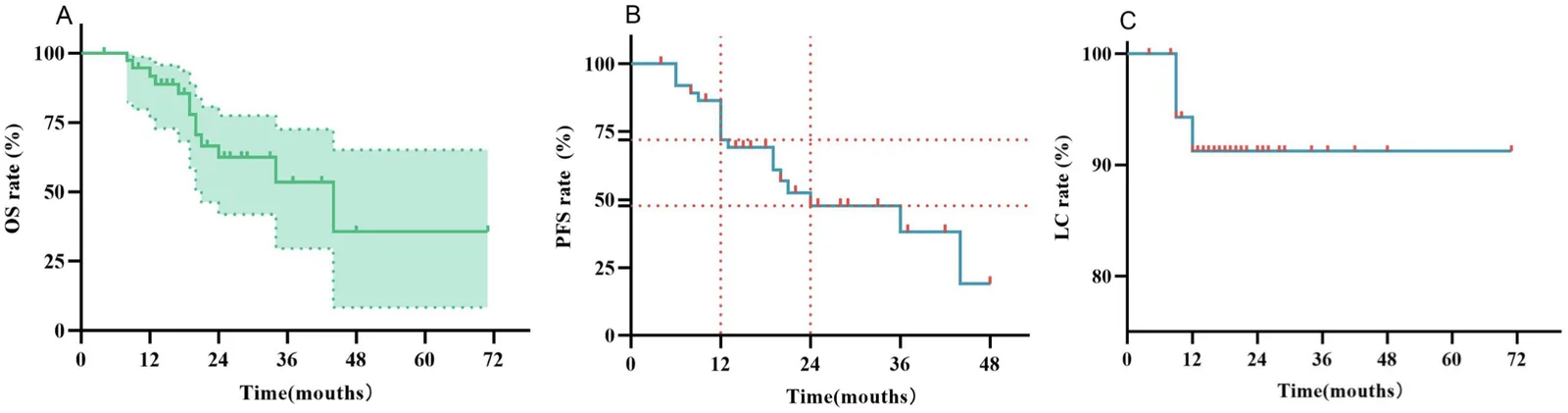
Survival curves of patients with limited-stage small-cell lung cancer treated with carbon-ion radiotherapy. **(A)** Overall survival (OS) curve; **(B)** Progression-free survival (PFS) curve; **(C)** Local control (LC) curve.

### 3.3 Dosimetric characteristics of radiotherapy plans

A total of 39 patients with limited-stage small-cell lung cancer were enrolled in this study, all of whom received carbon-ion radiotherapy. The target coverage and dosimetric parameters of key organs-at-risk (OARs) including the lung, esophagus, heart and spinal cord were systematically evaluated. **Figure 1** shows the transverse isodose distribution and dose-volume histogram (DVH) of a representative patient. Panel A is the transverse isodose cloud map, and Panel B is the corresponding DVH curve, which intuitively demonstrates the physical property that the high-dose region is concentrated inside the target volume and the dose drops rapidly outside the target **Figure 2**.

**Figure 2:**
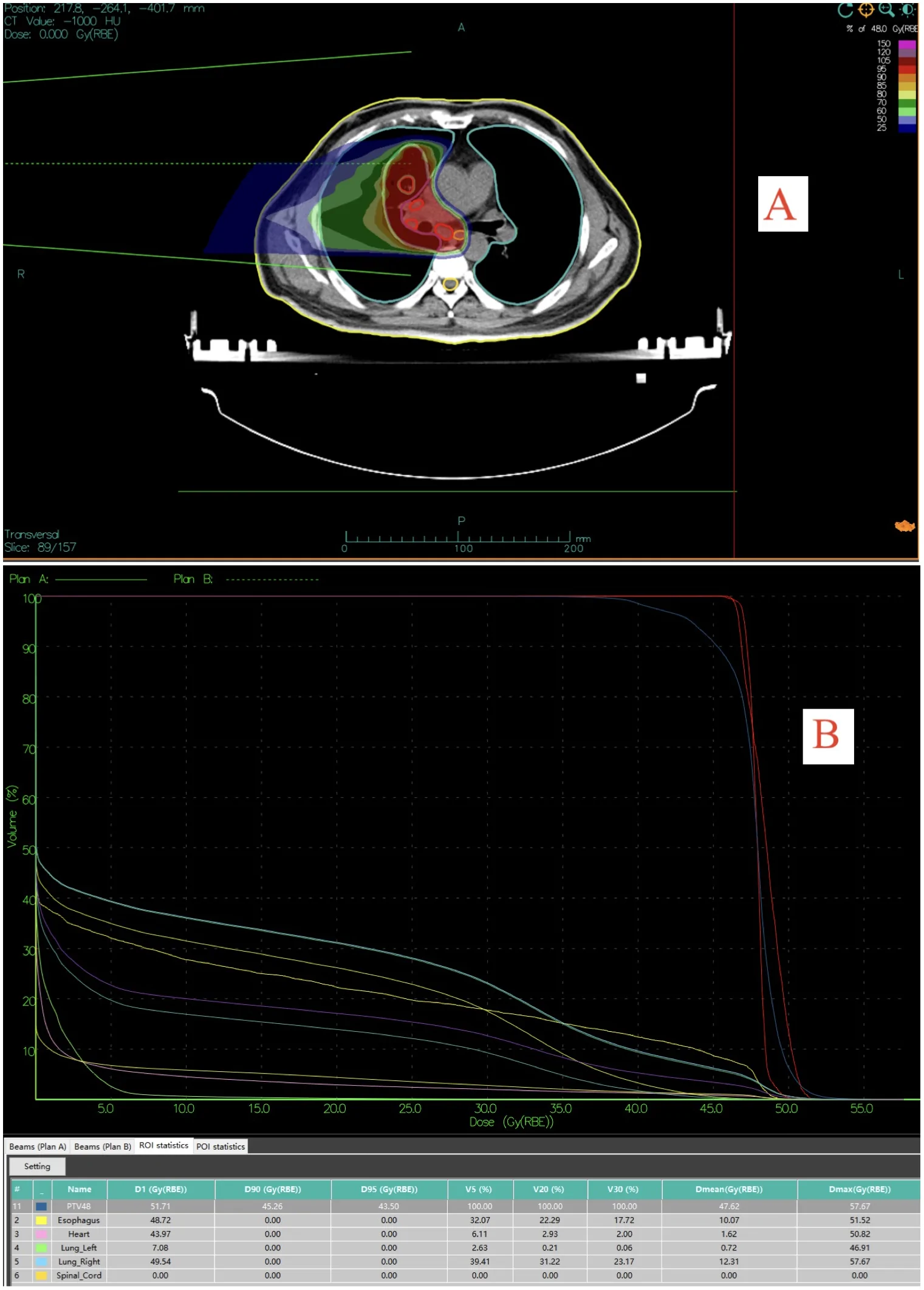
Transverse-plane dose distribution and dose-volume histogram (DVH) of carbon-ion radiotherapy plan for limited-stage small-cell lung cancer [**(A)** transverse-plane isodose cloud map; **(B)** DVH plot].

Dosimetric statistical results showed that the median volumes of GTV, CTV and PTV in this cohort were 66.0 mL, 275.5 mL and 419.6 mL, respectively. Target-volume coverage met the pre-set dose-volume constraint (V95 ≥ 90%) in all cases. The detailed dosimetric parameters (mean ± standard deviation) of each organ-at-risk are summarized in **Table 2**: for the left lung, D _mean_ was (5.15 ± 1.37) Gy(RBE), V5, V20, V30 were (18.67 ± 5.35)%, (12.09 ± 3.11)%, (11.75 ± 3.35)%;respectively; for the right lung, D _mean_ was (8.43 ± 1.28) Gy(RBE), V5, V20, V30 were (30.78 ± 4.28)%, (21.07 ± 2.01)%, (13.51 ± 2.29)%;respectively; for the heart, D _max_ (44.16 ± 4.00) Gy (RBE), D _mean_ (2.58 ± 0.68) Gy (RBE), V30 (3.94 ± 1.35) %, V40 (3.60 ± 1.70) %; D _max_ of the esophagus was (44.46 ± 2.50) Gy (RBE); D _max_ of the spinal cord was (4.38 ± 0.57) Gy (RBE) (**Table 2**). Among them, V20 of the affected-side lung exceeded the guideline-recommended limit in 13 patients, with a maximum value of 34.45%.

**Table 2:** Dosimetric parameters of organs-at-risk ( $\bar{X}\pm \text{S}$).

Organ-at-risk	Dosimetric parameter
Left lung	
D _mean_ [Gy (RBE)]	5.15 ± 1.37
V5(%)	18.67 ± 5.35
V20(%)	12.09 ± 3.11
V30(%)	11.75 ± 3.35
Right lung	
D _mean_ [Gy (RBE)]	8.43 ± 1.28
V5(%)	30.78 ± 4.28
V20(%)	21.07 ± 2.01
V30(%)	13.51 ± 2.29
Heart	
D _mean_ [Gy (RBE)]	2.58 ± 0.68
D _max_ [Gy (RBE)]	44.16 ± 4.00
V30(%)	3.94 ± 1.35
V40(%)	3.60 ± 1.70
Esophagus	
D _max_ [Gy (RBE)]	44.46 ± 2.50
Spinal cord	
D _max_ [Gy (RBE)]	4.38 ± 0.57

D mean, mean dose; D max, maximum dose; Vx, percentage of organ volume receiving ≥X Gy (RBE). Gy (RBE) denotes relative-biological-effectiveness-weighted dose.

### 3.4 Univariate analysis

To further explore prognostic factors for patients, 18 clinical and laboratory variables were incorporated into the univariate Cox proportional-hazards regression model, with overall survival (OS) and progression-free survival (PFS) set as outcome indicators, respectively. The hazard ratio (HR), 95% confidence interval (95% CI) and *P*-value for each variable are shown in **Table 3**. Univariate analysis demonstrated that none of the included variables reached the conventional statistical significance level (all *P*>0.05). Marginally significant trends (*P* < 0.10) were observed for KPS score with respect to OS (*P* = 0.085) and for NLR with respect to PFS (*P* = 0.084). Referring to previous studies on cancer prognosis, univariate *P*<0.15 was adopted as the cut-off value for selecting candidate variables into the multivariate model. For OS, KPS score and Caprini score entered the candidate pool. For PFS, NLR (*P* = 0.084) was selected as a candidate variable. Gender and smoking history with well-recognized clinical prognostic value, as well as KPS and Caprini scores, were included in the multivariate model for adjustment. No association was detected between remaining variables (*P*>0.15) and OS in univariate analysis.

**Table 3:** Univariate Cox regression analysis of patients stratified by different clinical parameters.

Variable	Category	N (%)	HR (95% CI) OS	P_OS	HR (95% CI) PFS	P_PFS
Gender	Female	8 20.5)	1.00 Reference)	0.648	1.00 Reference)	0.185
	Male	31 79.5)	1.42 0.31~6.52)		2.72 0.62~11.93)	
Age	≤65	25 64.1)	1.00 Reference)	0.723	1.00 Reference)	0.641
	>65	14 35.9)	0.81 0.26~2.53)		0.80 0.31~2.07)	
Alcohol	No	23 59.0)	1.00 Reference)	0.510	1.00 Reference)	0.340
	Yes	16 41.0)	1.46 0.47~4.49)		1.59 0.62~4.09)	
Comorbidity	No	20 51.3)	1.00 Reference)	0.503	1.00 Reference)	0.690
	Yes	19 48.7)	0.69 0.23~2.07)		1.21 0.48~3.07)	
Smoking	No	18 46.2)	1.00 Reference)	0.179	1.00 Reference)	0.370
	Yes	21 53.8)	2.26 0.69~7.43)		1.55 0.60~4.02)	
Caprini	<5	15 38.5)	1.00 Reference)	0.130	1.00 Reference)	0.239
	≥5	24 61.5)	4.90 0.63~38.29)		2.13 0.61~7.50)	
sleep	>6	28 71.8)	1.00 Reference)	0.240	1.00 Reference)	0.710
	≤6	11 28.2)	2.00 0.63~6.39)		1.22 0.42~3.53)	
BMI	≥24	20 51.3)	1.00 Reference)	0.378	1.00 Reference)	0.927
	<24	19 48.7)	0.61 0.20~1.83)		1.04 0.41~2.65)	
Tumor site	Left lung	19 48.7)	1.00 Reference)	0.320	1.00 Reference)	0.552
	Right lung	20 51.3)	0.56 0.18~1.76)		1.34 0.51~3.48)	
KPS	70~80	19 48.7)	1.00 Reference)	0.085	1.00 Reference)	0.252
	90	20 51.3)	0.32 0.09~1.17)		0.56 0.21~1.52)	
dose	≤68	20 51.3)	1.00 Reference)	0.954	1.00 Reference)	0.884
	>68	19 48.7)	1.03 0.35~3.09)		1.07 0.42~2.75)	
T	T1~T2	13 33.3)	1.00 Reference)	0.212	1.00 Reference)	0.817
	T3~T4	26 66.7)	0.49 0.16~1.49)		0.90 0.35~2.28)	
N	N0~N1	7 17.9)	1.00 Reference)	0.792	1.00 Reference)	0.672
	N2~N3	32 82.1)	0.81 0.18~3.77)		1.38 0.31~6.08)	
CRT sequence	Sequential	13 34.2)	1.00 Reference)	0.674	1.00 Reference)	0.313
	Concurrent	25 65.8)	1.29 0.40~4.21)		1.71 0.60~4.84)	
Chemo regimen	EP	22 57.9)	1.00 Reference)	0.451	1.00 Reference)	0.209
	EC	16 42.1)	1.53 0.51~4.58)		0.54 0.21~1.41)	
PCI	No	19 48.7)	1.00 Reference)	0.668	1.00 Reference)	0.250
	Yes	20 51.3)	1.28 0.42~3.92)		0.57 0.22~1.49)	
ICI	No	16 41.0)	1.00 Reference)	0.182	1.00 Reference)	0.437
	Yes	23 59.0)	2.47 0.65~9.32)		1.50 0.54~4.13)	
NLR	≤3	30 76.9)	1.00 Reference)	0.786	1.00 Reference)	0.084
	>3	9 23.1)	1.20 0.32~4.45)		2.76 0.87~8.77)	

*P* < 0.05 was considered statistically significant.

Candidate variables screened from univariate analysis including KPS score and Caprini score, together with gender and smoking history with well-recognized clinical prognostic value, were used to construct the multivariate regression model. Preliminary test of the proportional-hazards assumption revealed that NLR violated the proportional-hazards (PH) assumption (*P* = 0.040), which did not satisfy the prerequisites for standard Cox regression. Therefore, stratified Cox regression with NLR as the stratifying variable was adopted for OS analysis. Stratification was used to control the confounding effect of NLR on survival outcomes without estimating the hazard ratio (HR) effect of NLR itself, and the independent prognostic effects of other covariates were evaluated after adjusting for this variable. For the PFS outcome, NLR complied with the proportional-hazards assumption, and the standard Cox proportional-hazards regression was performed directly. Multivariate results are shown in **Table 4**.

**Table 4:** Multivariate cox regression analysis for OS and PFS.

Variable	Category	OS HR (95% CI)	P (OS)	PFS HR (95% CI)	P (PFS)
Gender	Male vs Female	0.589 (0.110-3.146)	0.535	2.143 (0.417-11.014)	0.361
Smoking	Yes vs No	1.522 (0.383-6.052)	0.551	0.890 (0.270-2.937)	0.848
**KPS**	**90 vs 70~80**	**0.203 (0.043-0.956)**	**0.044**	0.640 (0.228-1.801)	0.398
Caprini	≥5 vs<5	4.316 (0.426-43.737)	0.216	1.605 (0.365-7.059)	0.531
NLR	>3 vs ≤3	*Stratified*	–	2.554 (0.776-8.409)	0.123

OS model was stratified by NLR due to violation of proportional hazards assumption. PFS model was standard Cox regression. HR, hazard ratio; CI, confidence interval. Bold values indicate P < 0.05, statistically significant.

Results of stratified Cox regression for OS showed that KPS score was an independent protective factor for OS (HR = 0.203, 95% CI: 0.043-0.956, *P* = 0.044). Patients with a KPS score of 90 had a 79.7 % reduction in mortality risk compared with patients with KPS scores of 70-80. Notably, KPS only showed marginal significance in univariate analysis (*P* = 0.085), but reached statistical significance in the multivariate stratified Cox regression (*P* = 0.044), suggesting that the independent protective effect of KPS became evident after controlling for the confounding effects of NLR and other covariates. A Caprini score ≥5 (HR = 4.316, 95% CI: 0.426-43.737, *P* = 0.216) and smoking history (HR = 1.522, 95% CI: 0.383-6.052, *P* = 0.551) only exhibited a trend toward poor prognosis without statistical significance. No significant independent association was observed between gender and OS (HR = 0.589, 95% CI: 0.110-3.146, *P* = 0.535). The C-index of this stratified model was 0.691, indicating acceptable discriminatory power. A global test of the proportional-hazards assumption was performed for covariates other than the stratifying variable, yielding a global χ^2^ = 7.819, df=4, *P* = 0.098. No evidence of violation of the proportional-hazards assumption was found among the remaining covariates. Multivariate results from standard Cox regression for PFS demonstrated that none of the included variables showed independent predictive value for PFS (all *P*>0.05). The global test verified that the overall model satisfied the PH assumption (global χ^2^ = 5.269, df=5, *P* = 0.38).

### 3.5 Trends of serum tumor markers before and after treatment

Thirty-two patients had complete test results for NSE, CEA, CYFRA21-1, CA-125 and SCC, while complete proGRP measurements were available in 12 patients.

The median pre-treatment NSE level was 12.70 (7.30, 24.80) ng/mL, and the post-treatment level was 10.0 (7.20, 12.78) ng/mL. The paired *t*-test revealed a significant post-treatment decrease (mean decrease: 9.819 ng/mL, 95% CI: −15.72~−3.917, *t* = 3.393, df=31, *P* = 0.0019). Pre-treatment CEA was 3.20 (2.00, 5.10) ng/mL versus post-treatment 2.60 (1.57, 3.28) ng/mL, with a mean decrease of 1.424 ng/mL (95% CI: −2.747~0.1020, *t* = 2.197, df=31, *P* = 0.0356), showing a statistically significant difference. Pre-treatment CYFRA21-1 was 2.78 (1.70, 4.09) ng/mL and post-treatment was2.14 (1.78, 3.09) ng/mL, presenting a significant reduction (mean decrease: 0.9425 ng/mL, 95% CI: −1.582~−0.3034, *t* = 3.008, df=31, *P* = 0.0052). Pre-treatment CA-125 was 13.50 (8.10, 18.90) U/mL, post-treatment CA-125 was 11.75 (9.13, 18.88) U/mL, mean decrease 1.506 U/mL (95% CI: −7.236~4.224, *t* = 0.5361, df=31, *P* = 0.5957), with no statistically significant difference. Pre-treatment SCC was 1.60 (1.19, 2.20) ng/mL and post-treatment SCC was 1.56 (1.10, 2.17) ng/mL, mean decrease 0.5506 ng/mL (95% CI: −1.073~0.02836, *t* = 2.150, df=31, *P* = 0.0394), with a statistically significant difference. Pre-treatment proGRP was 196.5 (56.88, 422.00) pg/mL, and post-treatment proGRP was 46.8 (34.00, 81.15) pg/mL. Although proGRP showed a decreasing trend from baseline after treatment, the difference was not statistically significant (mean decrease: 424.0 pg/mL, 95% CI: −916.9~69.02, *t* = 1.893, df=11, *P* = 0.0850), as shown in **Figure 3**.

**Figure 3:**
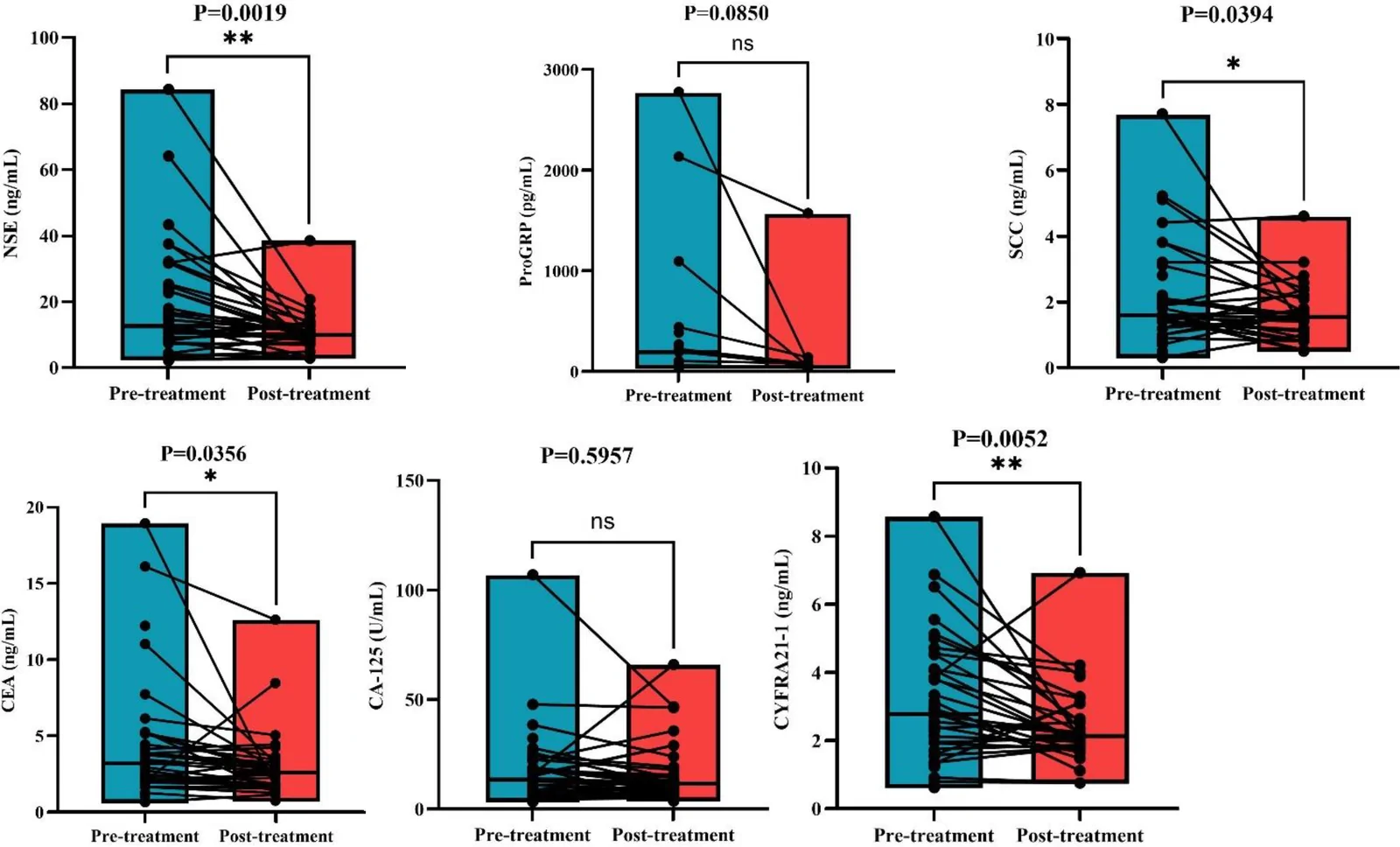
Comparison of serum tumor markers before and after carbon-ion radiotherapy. *P<0.05, **P<0.01, ns, not statistically significant.

To evaluate the prognostic predictive value of changes in tumor markers before and after carbon-ion radiotherapy, the change values of six tumor markers (Δ = post-treatment value − pre-treatment value; a negative value indicates a post-treatment decrease) were separately incorporated into the univariate Cox proportional-hazards regression model, with overall survival (OS) and progression-free survival (PFS) set as outcome indicators, respectively (**Table 5**). ProGRP was not included in this regression analysis because complete paired test data were available for only 12 patients with an insufficient sample size. Analysis for the OS outcome showed that the change in SCC was significantly associated with OS (HR = 0.640, 95% CI: 0.459-0.892, *P* = 0.0085), suggesting that a greater post-treatment decrease in SCC corresponded to a lower risk of death. The change in CYFRA21-1 showed a marginally significant protective trend (HR = 0.731, 95% CI: 0.524-1.019, *P* = 0.0647). No statistically significant correlations were observed between changes in NSE, CEA, CA-125 and OS (all *P*>0.05).

**Table 5:** Association analysis between changes in tumor markers before and after treatment and survival prognosis.

Tumor marker	N	OS HR (95% CI)	P (OS)	PFS HR (95% CI)	P (PFS)
**SCC**	32	**0.640 (0.459-0.892)**	**0.0085**	0.782 (0.581-1.053)	0.1052
CYFRA21-1	32	0.731 (0.524-1.019)	0.0647	**0.766 (0.596-0.983)**	**0.0364**
NSE	32	0.984 (0.952-1.017)	0.3375	0.982 (0.962-1.015)	0.3891
CEA	32	1.007 (0.900-1.126)	0.9074	0.952 (0.874-1.036)	0.2524
CA-125	32	0.989 (0.955-1.024)	0.5345	1.001 (0.972-1.031)	0.9285

Bold values indicate P < 0.05, statistically significant.

Analysis for the PFS outcome showed that the change in CYFRA21-1 was significantly associated with PFS (HR = 0.766, 95% CI: 0.596-0.983, *P* = 0.0364), suggesting that a more pronounced post-treatment decrease in CYFRA21-1 corresponded to a lower risk of disease progression. The change in SCC showed a protective trend without reaching statistical significance (HR = 0.782, 95% CI: 0.581-1.053, *P* = 0.1052). No significant associations were observed between changes in NSE, CEA, CA-125 and PFS (all *P*>0.05).

Univariate Cox regression analysis showed that the change in SCC was significantly associated with OS (HR = 0.640, 95% CI: 0.459-0.892, P = 0.0085), and the change in CYFRA21-1 was significantly associated with PFS (HR = 0.766, 95% CI: 0.596-0.983, P = 0.0364), suggesting that post-treatment decreases in these two tumor markers may be correlated with survival benefits for patients.

### 3.6 Safety

Adverse events observed during and after treatment mainly included radiation-induced esophagitis, radiation-induced pneumonitis, gastrointestinal reactions and myelosuppression, as shown in **Figure 4**. Radiation-related toxicities were as follows:8patients (20.5 %) developed radiation-induced esophagitis, including grade I in 7 (17.9 %) and grade II in 1 (2.6 %); 3 patients (7.7 %) had radiation-induced pneumonitis, including grade I in 2 (5.1 %) and grade II in 1 (2.6 %); one case (2.6 %) of radiation-induced pharyngeal edema was also noted. Myelosuppression occurred in 26 patients (66.7 %): grade I in 16 (41.0 %), grade II in 5 (12.8 %), grade III in 3 (7.7 %), and grade IV in 2 (5.1 %). Nausea and vomiting occurred in 6 patients (15.4 %); thrombocytopenia and leukopenia were observed in 10 patients (25.6 %). Gastrointestinal reactions were reported in 3 patients (7.7 %), including grade I in 2 (5.1 %) and grade II in 1 (2.6 %). The above-mentioned hematological toxicities and gastrointestinal reactions were mainly attributed to chemotherapy.

**Figure 4:**
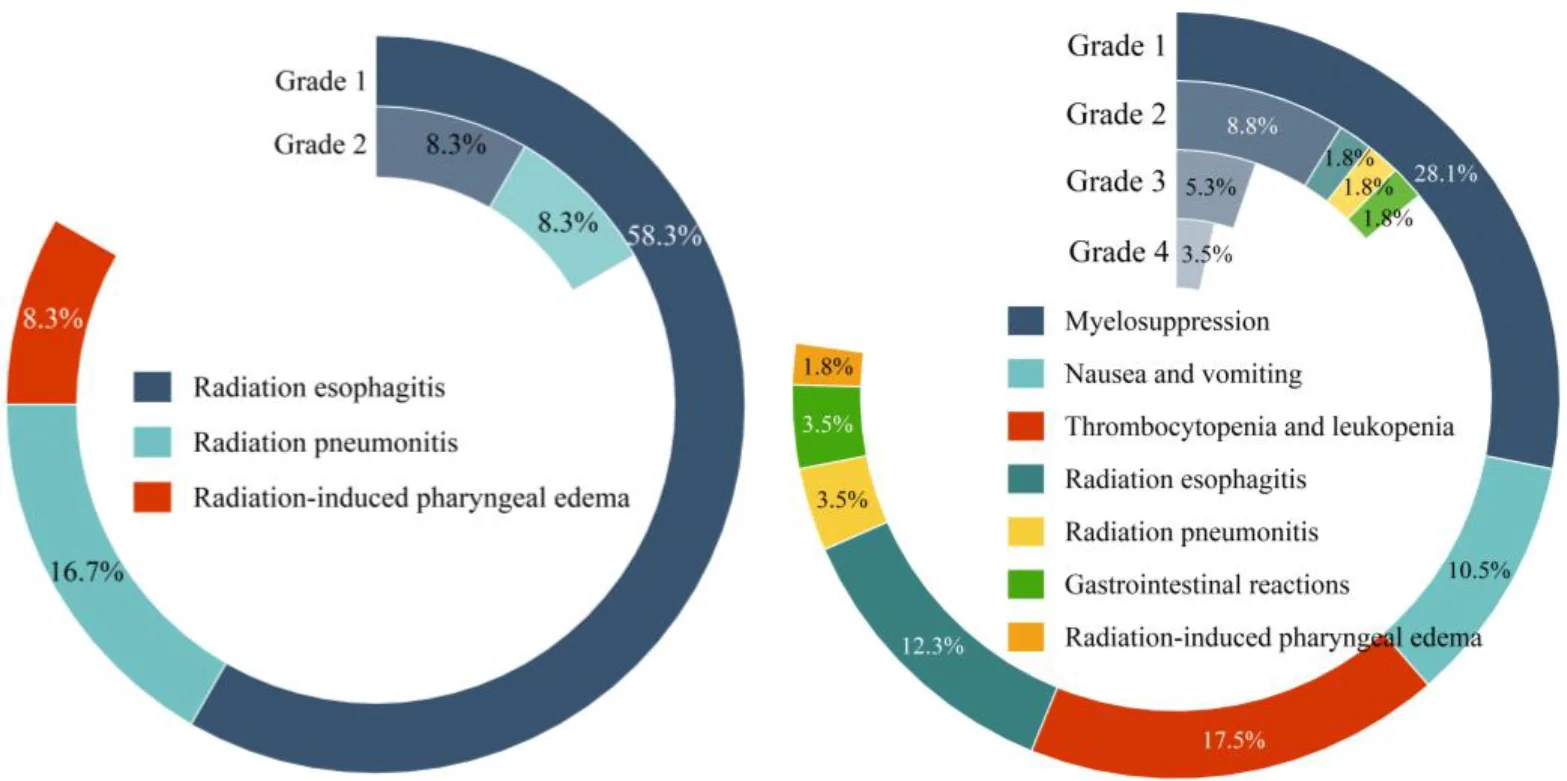
Adverse events in patients after carbon-ion radiotherapy. Left: composition ratio of radiation-related adverse events; Right: composition ratio of all chemoradiotherapy-related adverse events.

Radiation injuries directly associated with carbon-ion radiotherapy consisted of radiation-induced esophagitis, radiation-induced pneumonitis and radiation-induced pharyngeal edema, with an overall incidence of 30.8 %. All these radiation-related injuries were grade I-II, and no grade ≥3 radiation-associated injury was observed. Hematological and gastrointestinal adverse events were predominantly chemotherapy-derived. All adverse events were alleviated after symptomatic management (including acid suppression and gastric protection, antitussive and expectorant therapy, leukocyte/platelet-increasing agents, etc.). They did not significantly interfere with subsequent treatment procedures or patients’ quality of life, suggesting that carbon-ion radiotherapy combined with chemotherapy has an acceptable overall safety profile.

## 4 Discussion

Limited-stage small-cell lung cancer is highly malignant and aggressive. Although it is relatively sensitive to chemoradiotherapy, patients still face high risks of local recurrence and distant metastasis, and there remains a bottleneck in improving long-term survival (14). While conventional photon radiotherapy escalates the radiation dose to the tumor target volume, unavoidable irradiation to thoracic normal tissues such as the lung, esophagus and heart occurs. Severe radiation-related toxicities may reduce patients’ treatment tolerance and even interfere with treatment completion. Carbon-ion beams possess the physical advantage of the Bragg peak: high-dose deposition is concentrated within the tumor target volume, and the dose drops rapidly behind the target. This feature reduces the dose delivered to surrounding normal tissues while guaranteeing adequate target-volume irradiation. In addition, carbon-ion beams have a higher relative biological effectiveness (RBE) and exert cytotoxic effects against some radio-resistant tumor cells, which makes them a potential therapeutic modality for locally advanced thoracic tumors (15, 16).

This study adopted a simultaneous-integrated-boost dual-target carbon-ion radiotherapy strategy. Dose-boost irradiation was delivered to the primary tumor and involved lymph nodes, while prophylactic irradiation was administered to high-risk lymphatic drainage regions. The median volumes of GTV, CTV and PTV for the whole cohort were 66.0 mL, 275.5 mL and 419.6 mL, respectively. Target-volume coverage met the pre-set dose-volume constraint (V95 ≥ 90%) in all cases. Doses to other organs-at-risk including the heart, esophagus and spinal cord were mostly kept within the safety recommended thresholds specified in *Chinese Guidelines for Ion Therapy (2020 Edition) (*17).V20 of the affected-side lung exceeded the recommended limit in 13 patients in this group, with a maximum value of 34.45%, which was considered acceptable after comprehensive assessment combining patients’ baseline pulmonary function and anatomical relationship of target volumes. According to the dosimetric parameters of treatment plans in this cohort, carbon-ion radiotherapy could achieve adequate target-volume coverage, and doses for most organs-at-risk could be confined within guideline-recommended limits (18). Nevertheless, dosimetric advantages do not directly translate into clinical benefits. The correlation between dosimetric performance and survival outcomes still needs to be validated in larger-sample studies. Previous proton radiotherapy studies have improved partial pulmonary dosimetric parameters, but failed to confirm such improvements could be translated into survival benefits (19), indicating a translational gap between physical dose optimization and improved clinical efficacy.

In terms of clinical efficacy, all patients in this cohort successfully completed carbon-ion radiotherapy. The 3-month objective response rate was 64.10%; the 1- and 2-year local control rates were both 91.2%, and the 1- and 2-year overall survival rates were 91.65% and 62.45%, respectively. The median OS was 44 months, the 1- and 2-year progression-free survival rates were 72.00% and 47.77%, and the median PFS was 24 months. Descriptive cross-sectional comparisons of survival, local control and adverse event data were performed against published literature of various radiotherapy regimens (**Table 6**). A European-Canadian multicenter randomized trial (n = 547) reported 2-year OS rates of 56% with 45 Gy twice-daily versus 51% with 66 Gy once-daily (19). Relatively high numerical values of 1- and 2-year local control rates were observed in our cohort, which was hypothesized to be associated with the high biologically effective dose delivered to the tumor target volume. However, no statistical test for dose-effect correlation was performed in this study; therefore, a causal relationship between them could not be confirmed. Reduced dose to normal tissues theoretically facilitates treatment completion and may indirectly exert a positive effect on survival. Given substantial heterogeneity in enrollment baseline characteristics, chemotherapy regimens and follow-up periods across different studies, statistical tests were not conducted. Thus, we cannot directly conclude that carbon-ion radiotherapy is superior to other treatment modalities.

**Table 6:** Comparison of efficacy and safety among different treatment modalities.

Treatment modality	Author	N	Age (years)	Median survival	OS rate	LCR	Grade ≥3 adverse events
PBT	Rwigena et al., 2017 (28)	30	68 (57,81)	28.2	1-y:71.5%, 2-y:57.6%	1-y:85%, 2-y:69%	15(50%)
Conventional twice-daily (BID) radiotherapy	Luo J et al., 2023 (29)	100	60	33.6	1-y:92.0%, 3-y:45.7%	1-y:68.3%, 2-y:48.0%	35(35%)
AHF-TRT	M. Ono et al., 2023 (30)	13	67 (54,78)	–	1-y:100%, 3-y:76.9%, 5-y:53.9%	1-y:76.9%, 3-y:53.9%, 5-y:53.9%	0
Sintilimab plus concurrent chemoradiotherapy	X.Wang et al., 2022 (31)	20	63.5 (43,71)	–	1-y:90.0%, 2-y:69.3%	1-y:75%, 2-y:59.1%	9 (45%)
Hypofractionated radiotherapy	X.Y. Li et al., 2024 (32)	40	63 (52,80)	25	1-y:86.2%	1-y:79.5%	7 (17.5%)
Durvalumab combined regimen	W.B.Shen et al., 2024 (33)	43	–	32	1-y:87.7%, 2-y:67.1%, 3-y:49.1%	1-y:54.9%, 2-y:36.6%, 3-y:30.4%	14 (32.6%)

Further prognostic-factor analyses were performed. Univariate and multivariate Cox regression models were used to explore survival-related factors in patients with limited-stage small-cell lung cancer from two dimensions: clinical baseline characteristics and dynamic changes of post-treatment serum tumor markers. Eighteen clinical and laboratory baseline indicators were included for univariate Cox regression. None of these variables reached the conventional statistical significance threshold of *P*<0.05. KPS score showed a marginally significant trend for OS, and NLR showed a marginally significant trend for PFS, indicating their potential prognostic value. Referring to previous literature, variables for the multivariate model were selected based on univariate *P*<0.15 combined with clinical significance. The PH-assumption test revealed that NLR violated the proportional-hazards assumption. Therefore, stratified Cox regression with NLR as the stratifying variable was adopted for OS analysis to eliminate statistical bias caused by this variable. For the PFS outcome, NLR satisfied the proportional-hazards assumption, and standard Cox regression was applied. After multivariate adjustment for confounders, KPS score was identified as an independent protective factor for OS. Patients with a KPS score of 90 had a significantly lower risk of death compared with those with KPS scores of 70-80, suggesting that better physical status serves as a favorable independent predictor of overall survival in this cohort. This finding is consistent with several previous real-world cohort reports (20). Only trends toward poor prognosis were observed for Caprini thromboembolic risk score and smoking history, which became statistically non-significant after adjustment; hence they could not independently predict overall survival. No baseline variable included in this study was identified as an independent influencing factor for PFS, suggesting that disease progression in this cohort is driven by multiple interacting factors, and a single baseline indicator hardly enables independent prediction of progression-free survival.

Tumor markers serve as important reference indicators for evaluating tumor burden and treatment response in SCLC. In this study, paired serum marker levels before and after carbon-ion radiotherapy were compared. Significant post-treatment decreases from baseline were observed for NSE, CYFRA21-1, SCC and CEA, indicating that carbon-ion therapy could reduce systemic tumor burden. No statistically significant difference was found for CA-125 between pre- and post-treatment. Only 12 patients had complete paired ProGRP samples, leading to limited sample size and insufficient statistical power. The change-of-value Δ of each marker before and after treatment was incorporated into Cox regression for exploratory univariate prognostic analysis, while ProGRP was excluded from regression analysis due to sample-size constraints. Univariate results suggested that a greater magnitude of SCC reduction tended to correspond to better OS benefit, and a more pronounced decrease in CYFRA21-1 was associated with a favorable tendency for PFS benefit (21, 22). Given the limited sample size in this subsection, multiple-comparison correction was not performed. The prognostic predictive value of dynamic changes in tumor markers still needs to be further validated in external cohorts.

Safety results demonstrated that treatment-related acute adverse events in this cohort were predominantly grade I-II. No acute radiation-induced pneumonitis or esophagitis of grade ≥3 was observed. Hematological and gastrointestinal adverse events were mainly attributed to platinum-based combination chemotherapy. Grade 3-4 myelosuppression was chemotherapy-related and represented a key concern for this comprehensive treatment modality. Previous literature has reported a definite incidence of grade ≥2 radiation-induced pneumonitis and esophagitis among patients with limited-stage small-cell lung cancer receiving photon-based chemoradiotherapy (23, 24). Chemoradiotherapy combined with immunotherapy may also increase the potential risk of radiation-induced lung injury (25–27). Historical comparison with toxicity data from previous photon-based chemoradiotherapy showed a low incidence of severe radiation-related acute toxicities in our cohort, indicating that carbon-ion radiotherapy has promising value worth exploring in terms of thoracic normal-tissue protection. When evaluating the overall safety profile of carbon-ion radiotherapy, apart from local radiation-associated organ injuries, systemic adverse events induced by combined therapy should also not be ignored. Most hematological toxicities in this cohort were reversible after symptomatic intervention, showing acceptable hematological safety. It should be specially noted that only acute adverse events within 3 months were assessed in this study, and standardized data of late radiation-induced injuries were lacking. The risk of late toxicities after carbon-ion radiotherapy remains unclear and needs to be further clarified by standardized long-term follow-up.

This study has several notable limitations. It is a single-center, retrospective small-sample study, which inevitably introduces selection bias. The limited number of endpoint events constrains the statistical power of Cox regression, leading to wide confidence intervals for some variables. Some tumor marker assays were launched at a later time point, resulting in an insufficient number of paired specimens. The prognostic analysis of tumor markers was exploratory without correction for multiple comparisons. Only acute adverse events within 3 months after treatment were systematically collected in this study; follow-up data regarding late radiation-induced injuries are lacking, making it impossible to evaluate the late-toxicity profile of carbon-ion radiotherapy. In summary, the conclusions concerning efficacy, safety and prognosis derived from this study need to be further validated by multicenter, large-sample prospective cohorts, so as to further optimize the therapeutic strategies and prognostic evaluation system of carbon-ion radiotherapy for limited-stage small-cell lung cancer.

## 5 Conclusions

This study retrospectively analyzed 39 patients with limited-stage small-cell lung cancer (LS-SCLC) who received carbon-ion radiotherapy. Carbon-ion simultaneous-integrated-boost radiotherapy achieved favorable target-volume coverage, with satisfactory 1- and 2-year local control rates in this cohort. Acute radiation-related adverse events within 3 months after treatment were predominantly grade I-II, showing acceptable overall tolerance. It can serve as an alternative therapeutic option for locally advanced LS-SCLC patients with good general physical status. Multivariate analysis confirmed that KPS performance status was an independent protective factor for overall survival. Potential prognostic associations of post-treatment reductions in SCC and CYFRA21-1 were only observed in univariate analysis, which represented exploratory findings. As a single-center, small-sample retrospective study, this work suffers from selection bias. The above-mentioned results need to be further validated by multicenter cohorts with larger sample sizes.

## Data Availability

The original contributions presented in the study are included in the article/supplementary material. Further inquiries can be directed to the corresponding authors.
